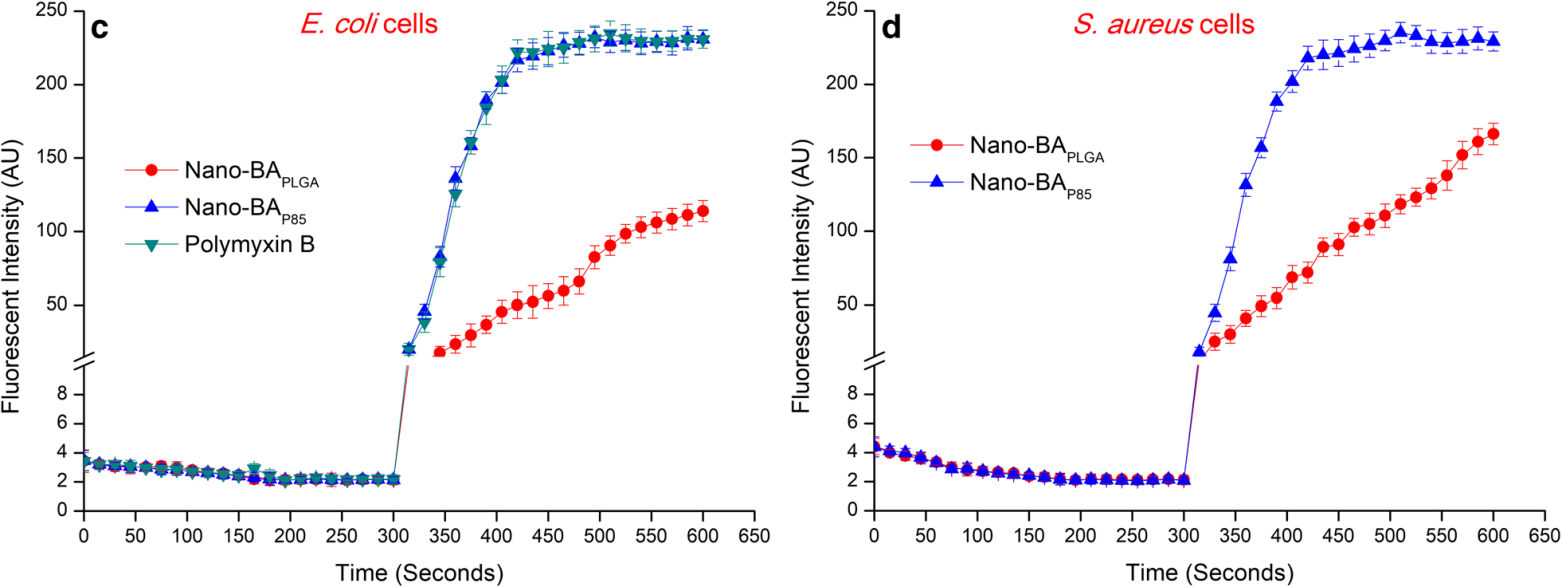# Correction to: Pluronic‑based nano‑self‑assemblies of bacitracin A with a new mechanism of action for an efficient in vivo therapeutic effect against bacterial peritonitis

**DOI:** 10.1186/s12951-019-0464-4

**Published:** 2019-03-06

**Authors:** Wei Hong, Lipeng Liu, Yining Zhao, Yinghui Liu, Dexian Zhang, Mingchun Liu

**Affiliations:** Key Laboratory of Zoonosis of Liaoning Province, College of Animal Science and Veterinary Medicine, Shenyang Agricultural University, Dongling Road 120, Shenyang, 110866 Liaoning People’s Republic of China

## Correction to: J Nanobiotechnol (2018) 16:66 10.1186/s12951-018-0397-3

In the original publication of the article [1], the figure panels 8c and d were published with incorrect values. The corrected figure panels 8c and d are given below: